# Fructose and Uric Acid: Major Mediators of Cardiovascular Disease Risk Starting at Pediatric Age

**DOI:** 10.3390/ijms21124479

**Published:** 2020-06-24

**Authors:** Elisa Russo, Giovanna Leoncini, Pasquale Esposito, Giacomo Garibotto, Roberto Pontremoli, Francesca Viazzi

**Affiliations:** 1 Clinica Nefrologica, Ospedale Policlinico San Martino, Dipartimento di Medicina Interna e Specialità Mediche, Università degli Studi di Genova, 16132 Genova, Italy; elisa24russo@gmail.com (E.R.); pasqualeesposito@hotmail.com (P.E.); gari@unige.it (G.G.); 2 Clinica di Medicina Interna 2, Ospedale Policlinico San Martino, Dipartimento di Medicina Interna e Specialità Mediche, Università degli Studi di Genova, 16132 Genova, Italy; giovanna.leoncini@unige.it (G.L.); roberto.pontremoli@unige.it (R.P.)

**Keywords:** fructose, uric acid, URAT-1, cardiovascular risk, children, metabolic syndrome, hypertension, NAFLD

## Abstract

Recently, there has been a growing interest in epidemiological and clinical studies supporting a pathogenetic role of fructose in cardio-metabolic diseases, especially in children and adolescents. In the present review, we summarize experimental data on the potential biological mechanisms linking fructose and uric acid in the development of insulin resistance, metabolic syndrome, obesity, diabetes, hypertension, non-alcoholic fatty liver disease and chronic renal disease, thereby contributing to an increase in cardiovascular risk at pediatric age.

## 1. Introduction

Fructose is one of the main sweetening agents in the human diet, and its consumption is rising worldwide. While monosaccharide fructose is naturally found in honey, fruit and vegetables, sugars added during the processing of food and drinks are the main source of fructose in the Western diet. In the United States, fructose extracted from corn starch is used to produce a syrup, called high-fructose corn syrup (HFCS), with a 42–55% fructose content. This compound has gradually replaced sugar in the United States dietary industry since the late 1960s mainly due to its low cost. In Europe, sucrose (a disaccharide derived by the association of fructose with glucose) is still the sweetener most used in food production [1]. The production of syrup of glucose and fructose in the European Union was limited by a regulation that, since October 2017, is no longer enforced. The production of HFCS is, therefore, expected to grow and to replace sucrose in certain food products, especially in liquid or semi-solid products such as soft drinks and ice creams [2].

Fructose-containing beverages or sugar-sweetened beverages (SSBs) are the most abundant and well characterized source of fructose in the diet [3]. The intestinal epithelium adsorbs fructose through glucose transport proteins (GLUT) 5 and 2; thereafter, the enzymes fructokinase, aldolase B and triokinase metabolize the fructose, mainly in the liver [2].

A large body of evidence has accumulated indicating a strong association between SSBs and obesity as well as related chronic diseases, especially in children [4,5]. In epidemiological studies, however, it may be difficult to differentiate the effects of fructose itself from those attributable to glucose.

The growing convergence of total fructose intake, elevation in serum uric acid and metabolic syndrome worldwide suggested that fructose-induced hyperuricemia may have a pathogenetic role in metabolic syndrome. Many organs and systems are involved, including the cardiovascular, endocrine, hepatic and endothelial systems, but not all the molecular and cellular action mechanisms of uric acid have yet been revealed. With the objective of reviewing the possible pathogenetic role of fructose and uric acid in the development of conditions that increase cardiovascular risk in children, we will assess and discuss the biological mechanisms underlying these associations.

## 2. Fructose Is Associated with Increased Uric Acid Production

Fructose is adsorbed by intestinal Glut5 transporter, while Glut2 and other transporters are involved in glucose transport in the liver (60 to 70%), kidney, adipose tissue, and other organs (30 to 40%). Most fructose is metabolized by fructokinase to fructose 1-phosphate. The phosphorylation of fructose results in the depletion of intracellular phosphate and ATP, with a transient inhibition of protein synthesis. The generation and degradation of adenosine monophosphate by adenosine monophosphate deaminase results in the synthesis of inosine monophosphate and uric acid. Uric acid increases in the cell and may transiently rise by 1 to 2 mg/dL in the circulation. The metabolization of fructose-1-phosphate by aldolase B generates acyl glycerol, diacylglycerol, glycogen and triglycerides [6]. Whereas glucose metabolism is regulated by the product inhibition of phosphofructokinase (through ATP and citrate), fructose is metabolized in an uncontrolled way to glycerophosphate and acetyl-CoA, which serve as substrates for triglyceride synthesis [7,8].

## 3. Insulin Resistance and Metabolic Syndrome

Fructose-rich SSB consumption, one of the strongest risk factors for the development of metabolic syndrome in adults [4,9], is associated with the development of pediatric insulin resistance too, and this relationship may partially be mediated by central adiposity and serum uric acid [10]. A novel mechanism for this association was described in adolescents with obesity and normal glucose tolerance. In these subjects, fructose but not glucose ingestion caused a rapid and sustained rise in insulin mediated by a significant rise in circulating GLP-1 levels (12-fold greater than in their lean peers) [11]. These findings are not consistently confirmed in adults [12], however. Interestingly, fructose consumption has been shown to hamper the anorexic signaling of insulin, leptin and GLP-1 [13], and therefore, the high levels of GLP-1 observed after fructose ingestion do not induce satiety in adolescents who are obese.

Fructose is especially effective at inducing weight gain, inducing leptin resistance and stimulating increased food intake. Furthermore, fructose-fed animals have been shown to develop high blood pressure, fatty liver, high serum triglycerides, and insulin resistance as compared to the control group. These effects have been described in fructose-fed animals as compared to the control groups, despite similar caloric intakes and body weights [14].

It has been shown in humans that fructose can induce visceral fat accumulation, dyslipidemia, insulin resistance, impaired postprandial fat oxidation, and reduced resting energy expenditure [15].

**Figure 1:**
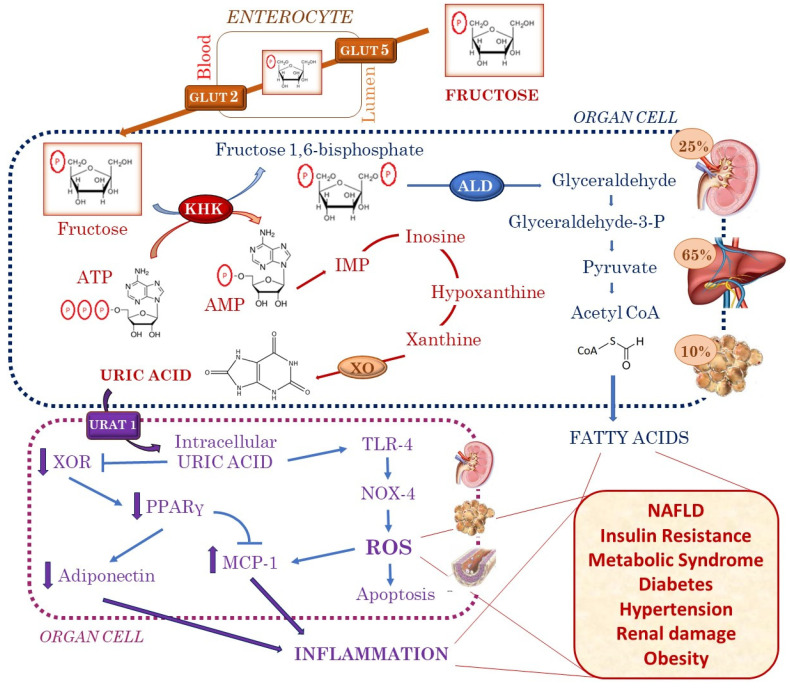
Fructose metabolism: direct effects and those mediated by uric acid. Glut 5 and Glut 2 guide fructose transport into cells where it is metabolized to fructose 1-phosphate by fructokinase (KHK). This reaction induces ATP depletion and causes the activation of AMP deaminase, purine degradation and uric acid generation. In addition, fructose generates glycerol phosphate and acetyl coenzyme A, resulting in fatty acid formation. Uric acid enters renal tubular cells, vascular muscle cells and adipocytes through a specific transporter, URAT-1, and activates NOX, resulting in ROS being generated from superoxide production. Moreover, uric acid can increase the production of MCP-1 and cause a decrease in the production of adiponectin, with a consequent pro-inflammatory response. Uric acid contributes to a pro-inflammatory state, mediated by toll-like receptor 4 with Nox4 up-regulation, promoting apoptosis in human cells. The production of fatty acids, oxidation, inflammation and pro-apoptotic pathways leads to several clinical manifestations, like NAFLD, insulin resistance and metabolic syndrome, diabetes, hypertension, obesity and renal damage, contributing to the increased cardiovascular risk from childhood. Abbreviations: GLUT 2, glucose transporter 2; GLUT 5, glucose transporter 5; KHK, fructokinase; ATP, adenosine triphosphate; AMP, adenosine monophosphate; IMP, inosine monophosphate; ALD, aldolase; XO, xanthine oxidase; XOR, xanthine oxidoreductase; PPARγ, peroxisome proliferator-activated receptor gamma; URAT1, uric acid transporter 1; ROS, reactive oxygen species; MCP 1, monocyte chemoattractant protein 1; Nox 4, NADPH oxidase 4; TLR 4, toll like receptor 4; NAFLD, non-alcoholic fatty liver disease.

Fructose is the only carbohydrate generating uric acid during its metabolism. In individuals eating fructose-rich meals, circulatory uric acid levels have been shown to rise within minutes of fructose ingestion and to remain elevated in the postprandial phase [16]. A great uptake and phosphorylation of fructose in the liver induces the depletion of intracellular adenosine triphosphate and leads to an increase in uric acid production [17] (Figure 1).

Uric acid is capable of inducing inflammation, oxidation [18] and endothelial damage and, therefore, might have a role in insulin resistance and metabolic syndrome [19]. It has been shown that lowering serum uric acid with either a xanthine oxidase inhibitor (allopurinol or febuxostat) or with the uricosuric, benzbromarone, could ameliorate the features of the metabolic syndrome in the fructose-fed rat. This contributes to the confirmation that serum uric acid might play a significant role in the link between fructose and the development of insulin resistance and metabolic syndrome [20]. Interestingly, serum uric acid and insulin are involved in a vicious cycle that causes the presence of one to induce an increase in the other, as shown by experiments demonstrating that physiological hyperinsulinemia acutely reduces urinary uric acid excretion in healthy volunteers and in essential hypertension [21,22].

In an experimental model of hyperuricemia and metabolic syndrome in mice with a leptin receptor defect, allopurinol inhibited macrophage infiltration and the decrease in adipose and circulating adiponectin, and improved blood pressure and insulin resistance [23].

## 4. Obesity 

The World Health Organization (WHO) reports childhood obesity to affect 35 million children in developed countries and that this feature is growing. Furthermore, overweight and obese children are more likely to develop diabetes and cardiovascular diseases early in their life [24,25]. An increase in the prevalence in obesity and diabetes closely parallels the dramatic increase in sugar dietary intake, especially that of fructose, due to the rising consumption of SSBs worldwide [26].

The American Heart Association recommends consuming no more than 100 to 150 kcal/day from all added sugar for most adults, and the World Health Organization and 2015 Dietary Guidelines Advisory Committee recommend limiting the intake of added sugars to no more than 10% of energy. However, according to the NHANES (National Health and Nutrition Examination Survey), approximately one-half of the U.S. population consumes SSBs and 25% obtains at least 200 calories from these beverages, equivalent to 1–2 cans of soda [27]. In particular, according to recent surveys, SSBs make up up to 9.3–9.7% of total energy intake for US adolescents [28], up to 14% of total energy intake for UK adolescents [29], and an even greater amount in Asia [30,31]. As a matter of fact, higher SSB intake could be a marker of a globally unhealthy diet, and there is concern that HFCS increases the risk of obesity in children compared with other caloric sweeteners [32]. Nevertheless, a high intake of both fructose and HFCS by children and adults has been associated with a greater risk of obesity and metabolic syndrome in several reports [33]. While not all published meta-analyses have reported a statistically significant link between fructose intake and clinically relevant dys-metabolism, recent RCTs support a benefit of reducing SSB consumption on adolescent obesity, suggesting that to achieve long-term benefits, the intervention needs to be sustained over time [34,35].

Adipose tissue in some obese subjects displays pro-inflammatory activity and has been associated with obesity-related comorbidities in both adults and children [36]. In fact, obesity seems to affect immune system function by an increase in MCP-1 and a decrease in adiponectin production in the adipose tissue [37]. Accordingly, an increase in macrophage infiltration with the expression of the pro-inflammatory cytokine TNF-α was observed in obese mice [38]. All these changes contribute to low-grade inflammation and metabolic syndrome, including insulin resistance, hypertension and an overall increased cardiovascular risk that have been described to be related to obesity [39]. The demonstration that lowering uric acid induced a reduction in macrophage infiltration and TNF-α expression as well as improving insulin sensitivity and blood pressure in animal models contributes to the hypothesis that serum uric acid plays a pathogenetic role in the development of obesity and its complications [39].

In youth, serum uric acid increases progressively from an early age with body growth and reaches a plateau at around 15–17 years [40]. It has been shown to be significantly higher in overweight/obese children than in their normal-weight peers and to be of particular significance as a predictor of metabolically unhealthy status in young populations [41]. In fact, serum uric acid has been associated with an increased cardiovascular risk in light of its ability to induce endothelial dysfunction, in particular, through a reduction in nitric oxide production and anti-proliferative effects on the endothelium [42]. In particular, metabolic syndrome, insulin resistance and high serum uric acid were significant predictors of left heart dimensions and mass in obese children [43]. Serum uric acid seems to represent a useful indicator to identify children subject to cardio-metabolic risk, and its use has been proposed to discriminate between metabolically healthy and metabolically unhealthy obesity. Nevertheless, large-scale longitudinal studies are needed to confirm that uric acid is a marker of the metabolic phenotypes associated with harmful obesity [41].

## 5. Diabetes

Several epidemiological studies have shown that for each one-per-day serving of SSBs, the risk of type 2 diabetes increases by about 20% both in the USA and in Europe [44,45]. This is, at least in part, attributable to all the mechanisms described above by which fructose is associated with an increased risk of insulin resistance, metabolic syndrome and obesity.

Once again, we would like to highlight the relationship between fructose and increased serum uric acid and lipid dysregulation [46]. Fructose and glucose require different enzymes in the initial steps of their metabolism. The phosphorylation process driven by fructokinase has no negative feedback system and results in intracellular ATP depletion, the activation of AMP deaminase, and uric acid generation, which induce cellular damage (Figure 1). Ishimoto et al. [47] have provided compelling evidence concerning the importance of fructokinase C—which is expressed primarily in the liver, intestines and kidneys—in mediating the adverse effects of fructose.

As a matter of fact, epidemiological studies have reported a strong association between metabolic syndrome and gout and/or hyperuricemia [48], and elevated serum uric acid has been found to independently predict the development of type 2 diabetes in several studies [49,50]. Finally, not only are the intake of fructose and an increase in serum uric acid involved in the development of type 2 diabetes but they also confer an additive risk profile to people diagnosed with diabetes. In fact, hyperuricemia proved to be common in adolescents with type 2 diabetes and to be associated with an increased risk of incident hypertension and an elevated urinary albumin excretion rate over 7 years [51].

## 6. Hypertension

Significant efforts have been made to develop new models of hypertension that better reflect the environmental and dietary behaviors of modern society. High fructose intake is involved in enhancing sodium (Na) reabsorption in both the kidney and the intestine, which may contribute to the development of hypertension [52]. As a matter of fact, it has been reported that feeding rats with a high-fructose (HF) diet causes salt-sensitive hypertension in conjunction with a high-salt (HS) diet [53]. It has also been proposed that fructose feeding stimulates intrarenal RAS activity (in particular, renal PRR and renin expression), which activates renal Na+ transporters (NHE3 and NKCC2), leading to increased salt sensitivity [54]. Moreover, under conditions of high fructose intake, the proximal tubule appears to become sensitized to Ang II via a protein kinase C mechanism, leading to increased rates of sodium reabsorption [55]. It has been recently reported that the adult offspring rats of mothers exposed to a 60% HF diet during pregnancy and lactation developed hypertension [56]. Accordingly, preclinical studies have shown that even the moderate consumption of fructose and sodium can significantly raise blood pressure [57].

These mechanisms, finally leading to an increase in blood pressure, have been hypothesized to be, at least in part, related to the increase in intracellular uric acid, one of the major metabolic end products of fructose (Figure 1). Uric acid has been shown to be able to stimulate local RAS with vascular smooth cell proliferation in rats and human vascular endothelial cell dysfunction, thus contributing to hypertension [58]. Moreover, uric acid induces the generation of intracellular reactive oxygen species via the nicotinamide adenine dinucleotide phosphate oxidase–dependent mitochondrial oxidant system and stimulates mitogen-activated protein kinase signaling, which, in turn, leads to the increased production of transforming growth factor [59]. Large studies have consistently confirmed the association between increased uric acid levels and hypertension in children and adolescents [60,61]. Moreover, uric acid has been shown to be a reliable predictor of future hypertension. Its active role as a determinant of blood pressure behavior throughout life at pediatric age has been confirmed by the fact it was associated with blood pressure values independently of anthropometric parameters of body composition and their variations [62]. In a large cohort of children at high cardiovascular risk, the presence of even moderately increased serum uric acid levels at baseline significantly blunted the decrease in blood pressure values observed in association with weight loss after lifestyle modifications during the study period. Therefore, serum uric acid assessment might help in the identification of those young patients in whom a higher vascular risk can be expected and signal a condition of hypertension more difficult to modulate [62].

The determinant role of uric acid in the pathogenesis of hypertension is confirmed by the demonstration that HF-induced renal pro-renin receptor and renin expression, as well as salt-sensitive hypertension, were significantly attenuated by allopurinol [54]. Moreover, two intervention studies showed allopurinol and uricosuric agents to be effective antihypertensive strategies in hyperuricemic children [63,64]. These findings taken together led some authors to propose hyperuricemia as a possible biomarker for the diagnosis of essential hypertension in children [65].

## 7. Renal Damage 

The possibility that fructose may have a role in the development of chronic kidney disease is gaining increasing attention, given its potential role in promoting hypertension and diabetes [66]. Indeed, the NHANES (1999–2004) reported that the intake of two or more sugar-containing beverages was associated with an increased risk of having albuminuria [26].

Fructose ingestion may also play a causal role in glomerular hypertension, renal inflammation and tubulointerstitial injury. Indeed, experimental studies support fructose intake being a mechanism for kidney injury. In fact, the administration of fructose (60% diet) to normal rats has been shown to induce renal hypertrophy, with tubular cell proliferation and low-grade tubulointerstitial injury [67], and to exacerbate proteinuria, worsen renal function, and accelerate glomerulosclerosis in a remnant kidney model [68]. A role for endogenous fructose has already been elucidated in diabetic nephropathy and in dehydration-mediated chronic kidney disease [69,70]. Furthermore, a relationship between endothelial dysfunction, thrombosis and fructose metabolism has recently been emphasized. Interestingly, the lack of fructokinase protects against the development of aging-associated kidney damage both in animals and humans [71].

Interestingly, the renal damage associated with fructose metabolism mimics what is observed in hyperuricemic rats and is inhibited by lowering uric acid [71]. The role of uric acid as a risk factor for the progression of renal function seems to be easier to detect when renal damage is in its initial phase. In a longitudinal study with 627 children and adolescents with mild to moderate chronic kidney disease (CKD) at presentation, it has been shown that a serum uric acid level > 7.5 mg/dL was an independent risk factor for a faster decline in renal function or initiation of renal replacement treatment [72].

We demonstrated that asymptomatic hyperuricemia is significantly associated with sub-clinical renal damage such as albuminuria and an increased intrarenal resistive index in essential hypertension [73]. Moreover, our studies contributed to the understanding of urate-induced tubular damage, suggesting that uric acid could work as damage associated molecular patterns (DAMPs) and produce an inflammatory and oxidative response by TLR4 engagement, which seems to be additive to the effects induced by angiotensin II [18]. Uric acid internalization by URAT 1 is necessary for its deleterious and pro-apoptotic effect at the tubular level, and this is confirmed by the protective effect showed by losartan, which reduced TLR4, MCP1 and Nox4 expression [74]. Taken together with the association between increased serum uric acid levels and the more severe tubular atrophy and vascular damage described in different series of renal biopsy [75,76,77,78,79,80], these findings might explain, at least in part, the unfavorable renal outcomes related to hyperuricemia.

Although large and well-designed RCTs will be needed to assess the precise effects of urate-lowering treatment on renal damage, recent intervention studies using XOis showed promising results in delaying CKD progression. The available results suggesting that hyperuricemia has a detrimental impact on kidney function are far from conclusive, and, therefore, it appears rational for clinicians to bear in mind that the treatment of so-called asymptomatic hyperuricemia to slow or delay the progression of CKD might be useful unless contraindicated [79].

## 8. NAFLD

Non-alcoholic fatty liver disease (NAFLD) is the hepatic manifestation of metabolic syndrome. NAFLD patients share laboratory and clinical features with people with metabolic syndrome, including high plasma triglycerides, low HDL cholesterol, impaired fasting glucose levels, an increased waist circumference and increased blood pressure [8]. Historically, NAFLD has been thought to result from overeating and a sedentary lifestyle. Its increasing prevalence parallels the increase in obesity and diabetes and seems to be linked to beverage consumption in young people [81].

An increased prevalence of pediatric obesity has been associated with a meaningful increase in NAFLD that has become the major cause of chronic liver disease in childhood in the last 20 years [82]. It has been widely recognized that NAFLD is associated with greater overall mortality [83] and that it represents an independent risk factor for cardiovascular diseases (CVDs) in adulthood. While this association is still debated in youth [84,85], recent data have shown that NAFLD could be considered as a marker of subclinical atherosclerotic damage as well as a strong cardiovascular risk factor, even at a very early age [86,87,88].

Fructose and the production of uric acid linked to its metabolism contribute to the pathogenesis of hepatic fat accumulation under normal and diseased states [89]. Fructose is directly lipogenic and also stimulates triglyceride synthesis via a purine-degrading pathway. Actually, the rapid phosphorylation of fructose by fructokinase generates AMP, which enters the purine degradation pathway through the activation of AMP deaminase, resulting in urate production (Figure 1). Uric acid induces the generation of mitochondrial oxidants leading to de novo lipogenesis. The metabolism of fructose in the intestine results in the disruption of the tight junctions and is likely to be responsible for the increased gut permeability that has been observed with fructose ingestion. Moreover, it results in endotoxins entering the portal vein, which is an important trigger for fatty liver formation and has been shown to be elevated in children with NAFLD [90].

Uric acid has also been shown to have other pro-inflammatory effects that could play a role in NAFLD, including the stimulation of the transcription factor NF-kB, an increase in chemokines such as monocyte chemoattractant protein-1, and the induction of inflammasomes [91]. Some studies indicate that dietary factors interfering with the gastrointestinal microbiota and microbial metabolism may be important in preventing or promoting NAFLD. In fact, the intestinal microbiota was shown to affect traits of the metabolic syndrome and some bacteria that do not possess the mannitol pathway for fructose metabolism, and to produce ethanol, which is known to induce liver steatosis and hyperuricemia [92,93].

A meta-analysis found an increase in the incidence of NAFLD by 3% for every 1 mg/dL rise in serum uric acid, even after accounting for the presence of potential confounding factors [94]. Finally, a large meta-analysis of 55,573 patients reported an increased occurrence of NAFLD (OR, 1.92; CI, 1.59–2.31) in subjects within the highest serum uric acid quintile as compared to those in the lowest [95]. NAFLD can also occur in subjects with normal and low BMI, and elevated uric acid is an essential feature in these patients [96].

From a clinical standpoint, the intake of SSBs is strongly linked to NAFLD. Reducing sugar or HFCS dietary intake may have major benefits for patients with NAFLD. Fructose-induced metabolic syndrome has been partially inhibited by treatment with xanthine oxidase inhibitors [20,97], with a documented reduction in the occurrence of fructose-mediated fatty liver [98]. Nevertheless, it is not possible to discriminate whether the protective role of allopurinol is mediated by reducing uric acid levels and/or by the effects of blocking xanthine oxidase-induced oxidative stress.

## 9. Why Are These Pathogenetic Mechanisms Extraordinarily Important in Children?

As reviewed above, fructose and uric acid may play a role in the development of high-risk conditions such as obesity, diabetes, hypertension, NAFLD and CKD, finally leading children and adolescents to an increased probability of developing cardiovascular disease ahead of time.

Children are an extraordinary model wherein mechanisms of disease can be investigated. In fact, potential confounding factors linked to co-morbidities or to the physiological aging are usually absent in the very early years of life. Moreover, it has been proposed that the pathogenetic mechanisms linking fructose and serum uric acid to the development of these high-risk conditions may follow a biphasic pattern: an initial phase mediated by vasoconstriction due to an angiotensin II increase and nitric oxide reduction and an organic, irreversible phase characterized by the proliferation and hypertrophy of vascular smooth muscle cells. This hypothesis might explain the conflicting results reported in studies investigating the use of urate- lowering treatments in adults with established hypertension or chronic renal disease. Thus, at variance with these data, small pilot studies conducted in children and adolescents showed a reduction in blood pressure [63,64] and improvement in insulin sensitivity [99,100] and CKD [101] with the use of urate-lowering treatments. We believe further studies are needed before definitive conclusions can be drawn regarding the reversibility of the mechanisms sustaining the relationship between fructose, uric acid and all the metabolic dysregulations.

## 10. Conclusions

In conclusion, we believe that the set of epidemiological and experimental data that we have summarized helps to clarify how an increase in fructose intake and the resulting increase in uric acid production contribute in a complex, multifactorial way to an increase in cardiovascular risk. Particularly, prospective and randomized studies conducted with adolescents will be able to clarify whether the modulation of these mechanisms, for example, with the use of urate-lowering treatments, could change the long-term cardiovascular prognosis in adulthood.
